# The cervical sagittal curvature change in patients with or without PCSM after laminoplasty

**DOI:** 10.3389/fsurg.2022.906839

**Published:** 2022-08-08

**Authors:** Shengjun Qian, Zhan Wang, Ying Ren, Ian Chew, Guangyao Jiang, Wanli Li, Weishan Chen

**Affiliations:** ^1^ Department of Orthopedic Surgery, The Second Affiliated Hospital, Zhejiang University School of Medicine, Hangzhou China; ^2^ Department of Orthopedic Surgery, Orthopedics Research Institute of Zhejiang University, Hangzhou China; ^3^ Department of Orthopedic Surgery, Zhejiang University School of Medicine, Hangzhou China

**Keywords:** PCSM, ligamentum flavum, laminoplasty, lordosis, kyphosis

## Abstract

**Objective:**

After laminoplasty, the cervical sagittal curvature of some patients tend to be lordotic, this phenomenon cannot be explained by the theory of laminoplasty, and the reason remains unknown. We explored the possible role played by pinching cervical spondylotic myelopathy (PCSM) in the cervical sagittal curvature change in patients after laminoplasty.

**Methods:**

From April 2017 to May 2019, we studied 122 patients undergoing laminoplasty with cervical spondylotic myelopathy (CSM). All patients were divided into Group A (anterior compression only, without PCSM) and Group B (both anterior and posterior compression, with PCSM). The visual analogue scale (VAS) was used to measure pain, and modiﬁed Japanese Orthopedic Association (mJOA) score was derived. The cervical global angle (CGA) and the range of cervical motion (ROM) were compared. The clinical and imaging results were compared between Group A and Group B.

**Results:**

After laminoplasty, both the mean VAS and mJOA scores improved significantly in Group A and Group B, the mJOA recovery rate of Group B was better than that of Group A (*P < 0.05*). The mean CGA and ROM decreased in Group A, but increased in Group B. MRI revealed that the ligamentum flavum of Group A was significantly thinner than that of Group B (*P < 0.05*).

**Conclusions:**

Because of the hypertrophic and folded ligamentum flavum compressing the dorsal spinal cord, patients with PCSM may maintain a compulsive kyphotic posture. After laminoplasty, the cervical sagittal curvature of these patients tend to be lordotic due to the release of dorsal spinal cord compression.

## Introduction

Laminoplasty is widely used to treat patients with cervical spondylotic myelopathy (CSM) in recent years (1, 2). Although laminoplasty protects the vertebral lamina, it still destroys the posterior ligament and paravertebral muscle, which results in postoperative neck pain and kyphotic cervical sagittal curvature (3). Similar to laminectomy, laminoplasty is also unsuitable for patients with kyphotic cervical sagittal curvature (4). However, recent studies have reported that the cervical sagittal curvature of some patients tend to be lordotic after laminoplasty, and the reason remains unknown (5).

Pinching cervical spondylotic myelopathy (PCSM) is a type of CSM with both anterior compression (herniated disc and osteophyte) and posterior compression (hypertrophic and folded ligamentum flavum). The ligamentum flavum is a member of the posterior ligamentous complex (PLC) located on the posterior edge of the spinal canal, which can reinforce the stability of the vertebrae (6). Many studies have found that a hypertrophic and folded ligamentum flavum can induce lumbar spinal canal stenosis and intermittent claudication (7–9). The neurological symptoms will be moderated when patients maintain a lumbar-flexed posture. We hypothesis that this situation may exist in patients with PCSM.

## Materials and methods

### Patient population

**Figure 1:**
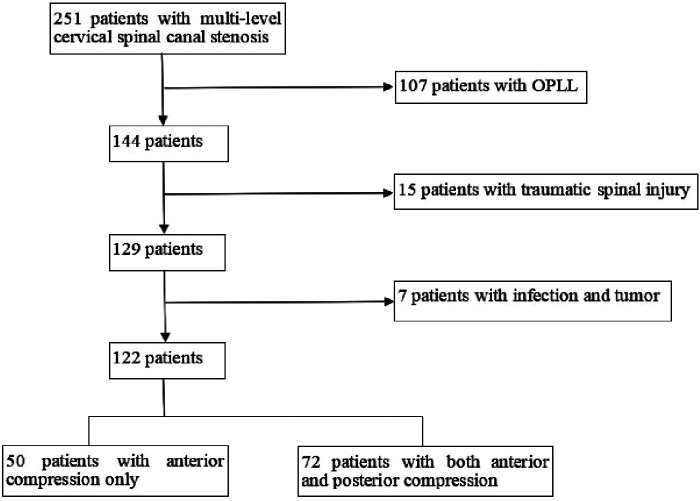
Flowchart of the collection of the study population.

**Figure 2:**
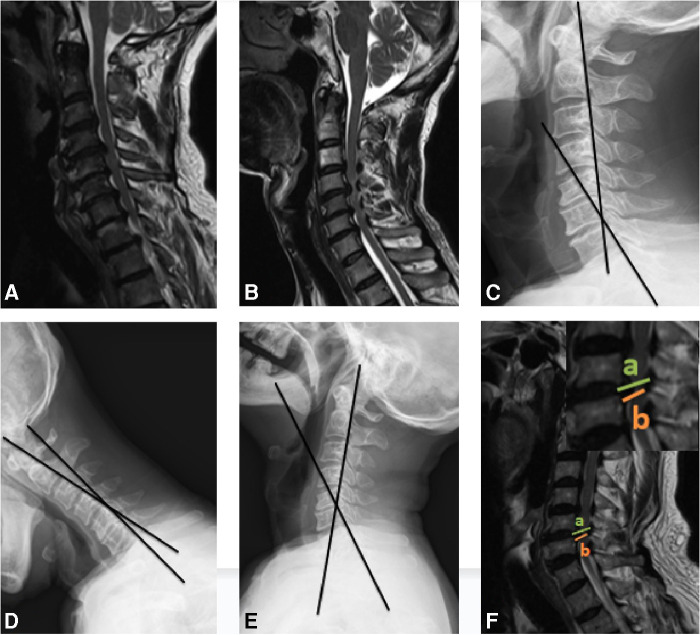
The schematic of Group A, Group B, CGA, ROM and thickness of ligamentum flavum. (**A**) Group A, anterior compression only, without PCSM. (**B**) Group B, both anterior and posterior compression, with PCSM. (**C**) The CGA is measured between the posterior border of the C2 and C7 vertebral body on neutral lateral radiograph. (**D,E**) The C2-7 ROM is calculated as the difference between the CGA measured during the maximal extension and ﬂexion on the dynamic lateral radiographs. (**F**) The thickness of ligamentum flavum was measured as the length of (a,b) at the site where the spinal cord was most seriously compressed according to sagittal MR (a was the distance of the anterior border and the posterior border of the spinal canal, b was the distance of the anterior border of the spinal canal and the posterior border of the spinal cord).

From April 2017 to May 2019, 251 patients undergoing laminoplasty with CSM in our hospital were analysed. Excluded from this study were 129 patients who had ossification of the posterior longitudinal ligament (OPLL), traumatic spinal injury, infection and tumor (Figure 1). The study enrolled a total of 95 men and 27 women with an average age of 59.0 years (range 38 to 79 years). Sixty five patients had three levels of cervical canal stenosis and 57 patients had four or five levels of cervical canal stenosis (Table 1). All patients were divided into two groups (Figures 2A,B): anterior compression only (Group A, 50 cases, without PCSM) and both anterior and posterior compression (Group B, PCSM, 72 cases, with PCSM). There was no significant difference in the factors of gender, age, BMI, basic diseases, intraoperative bleeding and surgical time between Group A and Group B (*P *> 0.05). The mean follow-up time was 27.32 months in Group A and 26.97 months in Group B.

**Table 1:** Clinical summary of 122 patients with CSM.

	Group A (*N* = 50)	Group B (*N* = 72)	*P-*value
Male, *N* (%)	37 (74.00)	58 (80.56)	0.391
Age, years, mean (SD)	58.82 (7.91)	59.24 (8.47)	0.261
BMI, kg/m^2^, mean (SD)	27.34 (4.11)	26.91 (3.94)	0.561
Diabetes, *N* (%)	6 (12.00)	9 (12.50)	0.934
Hypertension, *N* (%)	23 (46.00)	37 (51.39)	0.558
Stenotic segments			
3 segments, *N* (%)	28 (56.00)	37 (51.39)	0.616
4–5 segments, *N* (%)	22 (44.00)	35 (48.61)	0.616
Intraoperative bleeding, ml, mean (SD)	211.34 (108.76)	232.89 (122.63)	0.320
Surgical time, min, mean (SD)	102.62 (36.42)	109.13 (30.92)	0.290
Follow-up time, mon, mean (SD)	27.32 (3.55)	26.97 (3.45)	0.587

CSM, cervical spondylotic myelopathy; BMI, body mass index.

### Surgical technique

All patients were treated with unilateral open-door laminoplasty from C3 to C7. The paravertebral muscle was detached from each laminae. During this procedure, the muscles attached to the C2 and C7 spinous process were preserved as far as possible. A high-speed air drill was used to open the hemilamina on the dominant symptomatic side. A shallow gutter was scored on the contralateral hemilamina and used as a hinge. After opening the laminae, the hinged laminae was fixed with a titanium miniplate, and small screws were drilled through the plate holes into the lateral mass and the open laminae. Two drainage tubes were placed before incision suture, and a cervical collar was used for 2–4 weeks after surgery.

### Outcome measures

Clinical outcomes were assessed using a visual analogue scale (VAS) and modiﬁed Japanese Orthopedic Association (mJOA) score. The VAS measures pain on a scale of 0 (no pain) to 10 (maximal pain). The mJOA score is a 17-point rating instrument that evaluates sensory functions (of the trunk, upper and lower extremities), motor functions (of the upper and lower extremities), and the urinary bladder function. The mJOA recovery rate is deﬁned as follows: mJOA recovery rate (%) = (postoperative mJOA−preoperative mJOA)/(17−preoperative mJOA) × 100%.

Cervical lateral radiographs (neutral, extension and ﬂexion) were obtained and magnetic resonance imaging (MRI) was performed. We used the cervical global angle (CGA) to measure the angle of cervical sagittal curvature, which was measured between the posterior borders of the C2 and C7 vertebral bodies (10). The C2–7 ROM was the difference between the CGAs measured during maximal extension and ﬂexion on dynamic lateral radiographs **(**Figures 2C–E). Ligamentum flavum thickness was measured at the site where the spinal cord was most seriously compressed as indicated by sagittal MRI (11) (Figure 2F). All measurements were performed three times by one of the authors and independently by an experienced musculoskeletal radiologist.

### Statistical analysis

T-tests, and chi-square tests were used for statistical analysis. The analysis was carried out by SPSS 20.0, and a *P* value of <0.05 was considered statistically significant.

## Results

### Clinical outcomes

The mean VAS score in Group A was 5.62 ± 1.76 before surgery and 3.18 ± 2.01 at the last follow-up. The mean VAS score in Group B was 5.57 ± 1.75 before surgery and 2.54 ± 1.46 at the last follow-up. The mean mJOA score in Group A was 9.06 ± 2.40 before surgery and 12.92 ± 2.69 at the last follow-up. The mean mJOA score in Group B was 9.32 ± 2.44 before surgery and 14.01 ± 2.08 at the last follow-up. Thus, both the mean VAS and mJOA scores improved significantly in Group A and Group B after surgery. Additionally, the VAS change in Group A was smaller than that in Group B (2.44 ± 1.15 vs. 3.03 ± 1.26, *P < 0.05*), the mJOA change in Group A was also smaller than that in Group B (3.86 ± 1.55 vs. 4.69 ± 1.94, *P < 0.05*). Correspondingly, the mJOA recovery rate of Group A was lower than that of Group B (0.52 ± 0.21 vs. 0.63 ± 0.19, *P < 0.05*; Table 2).

**Table 2:** Comparison of VAS, mJOA and the mJOA recovery rates between Group A and Group B.

	Group A	Group B	*P*-value
Pre-Op VAS	5.62 ± 1.76	5.57 ± 1.75	0.876
Post-Op VAS	3.18 ± 2.01	2.54 ± 1.46	0.058
VAS change	−2.44 ± 1.15	−3.03 ± 1.26	0.010
Pre-Op mJOA	9.06 ± 2.40	9.32 ± 2.44	0.562
Post-Op mJOA	12.92 ± 2.69	14.01 ± 2.08	0.013
mJOA change	3.86 ± 1.55	4.69 ± 1.94	0.013
mJOA recovery rate	0.52 ± 0.21	0.63 ± 0.19	0.005

VAS change = Post-Op VAS – Pre-Op VAS, mJOA change = Post-Op mJOA – Pre-Op mJOA. mJOA recovery rate (%) = (Post-Op mJOA−Pre-Op mJOA) / (17−Pre-Op mJOA) × 100%. Values are displayed as a mean ± standard deviation. Significance between the two groups, *P < 0.05*. VAS,visual analogue scale; mJOA, modiﬁed japanese orthopedic association.

### Radiologic assessments

The mean CGA of Group A was 19.52° ± 9.58° before surgery and 13.62° ± 9.74° at the last follow-up. The mean CGA of Group B was 17.49° ± 9.16° before surgery and 20.34° ± 8.35° at the last follow-up. The mean ROM of Group A was 45.68° ± 8.69° before surgery and 37.74° ± 8.01° at the last follow-up. The mean ROM of Group B was 39.56° ± 8.86° before surgery and 41.34° ± 8.81° at the last follow-up. Thus, for CGA, a kyphotic change (5.89° ± 4.22°) in Group A and a lordotic change (2.85° ± 6.24°) in Group B was observed (*P < 0.05*). For ROM, there was a decreased change (7.94° ± 3.15°) in Group A and an increased change (1.78° ± 6.32°) in Group B (*P < 0.05*). The different results may imply the different factors that influence the CGA and ROM before laminoplasty between Group A and B. MRI revealed that the ligamentum flavum of Group A was notably thinner than that of Group B (1.98 mm ± 0.43 mm vs. 3.42 mm ± 0.69 mm, *P < 0.05*, Table 3), indicating patients with PCSM had hypertrophic and folded ligamentum flavum.

**Table 3:** Comparison of CGAs, ROMs and ligamentum flavum thicknesses between Group A and Group B.

	Group A	Group B	*P-*value
Pre-Op CGA (°)	19.52 ± 9.58	17.49 ± 9.16	0.240
Post-Op CGA (°)	13.62 ± 9.74	20.34 ± 8.35	<0.001
CGA change (°)	−5.89 ± 4.22	2.85 ± 6.24	<0.001
Pre-Op ROM (°)	45.68 ± 8.69	39.56 ± 8.86	<0.001
Post-Op ROM (°)	37.74 ± 8.01	41.34 ± 8.81	0.023
ROM change (°)	−7.94 ± 3.15	1.78 ± 6.32	<0.001
Thickness of LF (mm)	1.98 ± 0.43	3.42 ± 0.69	<0.001

CGA change = Post-Op CGA – Pre-Op CGA, ROM change = Post-Op ROM – Pre-Op ROM. Values are displayed as a mean ± standard deviation. Significance between the two groups, *P < 0.05*. CGA, cervical global angle; ROM, range of cervical motion.

### Complication

Postoperative complications were noted during the study: such as incision infection (4.00% vs. 4.17%), hematoma (2.00% vs. 2.78%), cerebrospinal fluid leakage (2.00% vs. 1.39%), spinal cord injury (2.00% vs. 2.78%), persistent axial pain (12.00% vs. 11.11%), C5 paresis (6.00% vs. 4.17%) and postoperative thrombosis (2.00% vs. 2.78%) between Group A and Group B (Table 4). There were no statistical differences between Group A and Group B in postoperative complications.

**Table 4:** Postoperative complication between Group A and Group B.

	Group A	Group B
Incision infection, *N* (%)	2 (4.00)	3 (4.17)
Hematoma, *N* (%)	1 (2.00)	2 (2.78)
Cerebrospinal fluid leakage, *N* (%)	1 (2.00)	1 (1.39)
Spinal cord injury, *N* (%)	1 (2.00)	2 (2.78)
Persistent axial pain, *N* (%)	6 (12.00)	8 (11.11)
C5 paresis, *N* (%)	3 (6.00)	3 (4.17)
Postoperative thrombosis, *N* (%)	1 (2.00)	2 (2.78)

## Discussion

As first reported by Hirabayashi, laminoplasty is an effective treatment for patients with CSM (12). By enlarging the spinal canal volume, laminoplasty can provide direct posterior local decompression, by allowing the posterior migration of the spinal cord, laminoplasty can also create an indirect anterior decompression (13). However, it should be noted that laminoplasty destroys the posterior ligament and paravertebral muscle. Thus, in most patients, a decrease in cervical lordosis or an increase in cervical kyphosis occurs after laminoplasty (14, 15). Laminoplasty is thus suitable for patients with cervical lordosis, but not suitable for patients with cervical neutral or kyphosis (16). But, recent studies have reported that the cervical sagittal curvature of some patients tend to be lordotic after laminoplasty (17), and the reason remains unknown.

The ligamentum flavum is located on the posterior edge of the spinal canal, which reinforces the stability of cervical vertebrae (18). With age, the ligamentum flavum gradually degenerates, becoming hypertrophic and folded (19), reducing the volume of the cervical spinal canal and ultimately leads to the compression of the dorsal spinal cord. PCSM is defined as CSM with both anterior and posterior compression, and the posterior compression is usually due to the the hypertrophic and folded ligamentum flavum. Many previous studies found that a hypertrophic and folded ligamentum flavum could induce lumbar spinal canal stenosis (20). If the patient straightens the back, the hypertrophic and folded ligamentum flavum will compress the spinal cord or the nerve roots to a greater extent, inducing lower limb radiating pain and aggravating intermittent claudication. Conversely, the neurological symptoms will be relieved if the patient maintains a lumbar-flexed posture. We hypothesise that the situation may exist in patients with PCSM, who have to maintain a cervical kyphotic posture to relieve the compression from posterior hypertrophic ligamentum flavum (21–23).

**Figure 3:**
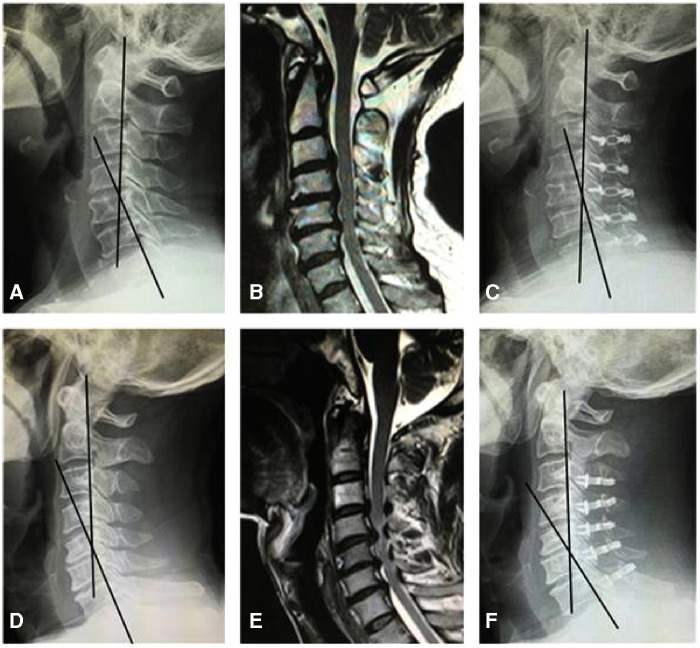
Radiological presentation of a 59 year-old man from Group A (**A–C**) and a 56 year-old man from Group B (**D–F**). (**A**) Preoperative lateral X-ray showed the CGA was 25.8°. (**B**) MR revealed the spinal cord had anterior compression only. (**C**) Postoperative lateral X-ray showed the CGA was 18.6°. (**D**) Preoperative lateral X-ray showed the CGA was 22.3°. (**E**) MR revealed spinal cord had both anterior and posterior compression. (**F**) Postoperative lateral X-ray showed the CGA was 31.8°.

From April 2017 to May 2019, we analysed 122 patients who were diagnosed with CSM and treated with laminoplasty in our hospital. All patients were divided into two groups: anterior compression only (Group A, without PCSM) and both anterior and posterior compression (Group B, with PCSM). The ligamentum flavum of Group A (1.98 mm ± 0.43 mm) was notably thinner than that of Group B (3.42 mm ± 0.69 mm). After over 2 years follow-up, for the CGA measurements, there was a kyphotic change (5.89° ± 4.22°) in Group A and a lordotic change (2.85° ± 6.24°) in Group B. This suggests that the cervical sagittal curvature change of Group A tends to be kyphotic after laminoplasty (Figures 3A–C), whereas that of Group B tends to be lordotic (Figures 3D–F). Moreover, the VAS and mJOA changes, and the mJOA recovery rate of patients in Group A, were significantly lower than those of patients in Group B, which suggests a greater improvement in the clinical results of patients in Group B, as compared to patients in Group A. Considering the thickness difference of ligamentum flavum between Group A and B, we conjecture that patients with PCSM had a forced kyphotic posture before laminoplasty. Neck extension was restricted due to the hypertrophic and folded ligamentum flavum. Compared with patients in Group A, the clinical and imaging results of patients in Group B seemed worse before laminoplasty. After laminoplasty, the patients' spinal canal were enlarged and the posterior compressions were no longer visible. This relieved patients of the forced kyphotic posture and allowed them to extend their neck freely. As such, the patients had higher levels of comfort. This corresponds with the improved results seen in Group B after laminoplasty.

Interestingly, compared with Group A (45.68° ± 8.69°), the mean preoperative ROM in Group B (39.56° ± 8.86°) was much smaller, implying that patients with PCSM had stiff necks. The theory of laminoplasty cannot account for the increased ROM (41.34° ± 8.81°) of Group B patients after laminoplasty, but is well explained by our hypothesis. Thus, the ROM may be useful when evaluating whether a patient has a compulsive kyphotic posture before laminoplasty.

Our study had certain limitations. First, this was a single-center study. Second, the number of cases was not large, and the follow-up time was short. As time goes on, a patent cervical spinal canal may appear in some patients, and leads to a compulsive kyphotic posture again. More cases will be included and the follow-up period will be lengthened in our future study.

## Conclusions

In conclusion, a hypertrophic and folded ligamentum flavum may force patients with PCSM to maintain a compulsive kyphotic posture. For these patients, the cervical sagittal curvature tend to become lordotic with the release of dorsal spinal cord compression after laminoplasty.

## Data Availability

The original contributions presented in the study are included in the article/**Supplementary Material**, further inquiries can be directed to the corresponding author/s.
